# Insulin Reduces Neuronal Excitability by Turning on GABA_A_ Channels that Generate Tonic Current

**DOI:** 10.1371/journal.pone.0016188

**Published:** 2011-01-14

**Authors:** Zhe Jin, Yang Jin, Suresh Kumar-Mendu, Eva Degerman, Leif Groop, Bryndis Birnir

**Affiliations:** 1 Department of Neuroscience, Uppsala University, Uppsala, Sweden; 2 Lund University Diabetic Centre, Lund University, Lund, Sweden; University of Cincinnatti, United States of America

## Abstract

Insulin signaling to the brain is important not only for metabolic homeostasis but also for higher brain functions such as cognition. GABA (γ-aminobutyric acid) decreases neuronal excitability by activating GABA_A_ channels that generate phasic and tonic currents. The level of tonic inhibition in neurons varies. In the hippocampus, interneurons and dentate gyrus granule cells normally have significant tonic currents under basal conditions in contrast to the CA1 pyramidal neurons where it is minimal. Here we show in acute rat hippocamal slices that insulin (1 nM) “turns on” new extrasynaptic GABA_A_ channels in CA1 pyramidal neurons resulting in decreased frequency of action potential firing. The channels are activated by more than million times lower GABA concentrations than synaptic channels, generate tonic currents and show outward rectification. The single-channel current amplitude is related to the GABA concentration resulting in a single-channel GABA affinity (EC_50_) in intact CA1 neurons of 17 pM with the maximal current amplitude reached with 1 nM GABA. They are inhibited by GABA_A_ antagonists but have novel pharmacology as the benzodiazepine flumazenil and zolpidem are inverse agonists. The results show that tonic rather than synaptic conductances regulate basal neuronal excitability when significant tonic conductance is expressed and demonstrate an unexpected hormonal control of the inhibitory channel subtypes and excitability of hippocampal neurons. The insulin-induced new channels provide a specific target for rescuing cognition in health and disease.

## Introduction

The insulin receptor is prominently expressed in the hippocampus suggesting that insulin regulates hippocampal function and thereby possibly modulates cognition [1]. Impaired insulin signaling increases risk of Alzheimer disease [2], cognitive disabilities in diabetes mellitus [3] and decreases cerebrocortical beta activity in overweight humans [4] whereas intranasal administration of insulin improves hippocampal-dependent memory function [5]. Nevertheless, the mechanism underlying the insulin effects on hippocampal function is not understood.

GABA, the main inhibitory neurotransmitter in the CNS binds to synaptic and extrasynaptic GABA_A_ channels that mediate phasic and tonic inhibition, respectively. The level of tonic inhibition in neurons varies [6], [7], [8], [9] and is dependent on the extracellular GABA concentration plus the GABA affinity of the channels in the neuronal plasma membrane. During exposure to novel environment or stress extracellular GABA concentrations may change [10] implying that GABA-activated tonic conductances are valuable under these circumstances. Accordingly, tonic inhibition in the hippocampus appears to modulate cognitive functions [11], [12], [13], [14], [15]. But, what determines subtypes and subcellular location of GABA_A_ channels and thereby the relative contribution of synaptic and extrasynaptic currents to neuronal function is still somewhat elusive.

Since the early 80's it has been known that insulin inhibits spontaneous firing of rat hippocampal pyramidal neurons [16] and in the 90's it was shown that insulin increases the number of synaptic GABA_A_ channels [17]. Whether insulin also affects the extrasynaptic GABA_A_ channels-mediated tonic inhibition in the hippocampus has not been examined. Here we show that the major effect of insulin on the hippocampal CA1 pyramidal neuronal excitability is achieved by increasing the GABA-mediated tonic inhibitory conductance.

## Results

### Insulin induces GABA_A_-mediated tonic current in hippocampal CA1 neurons

**Figure 1:**
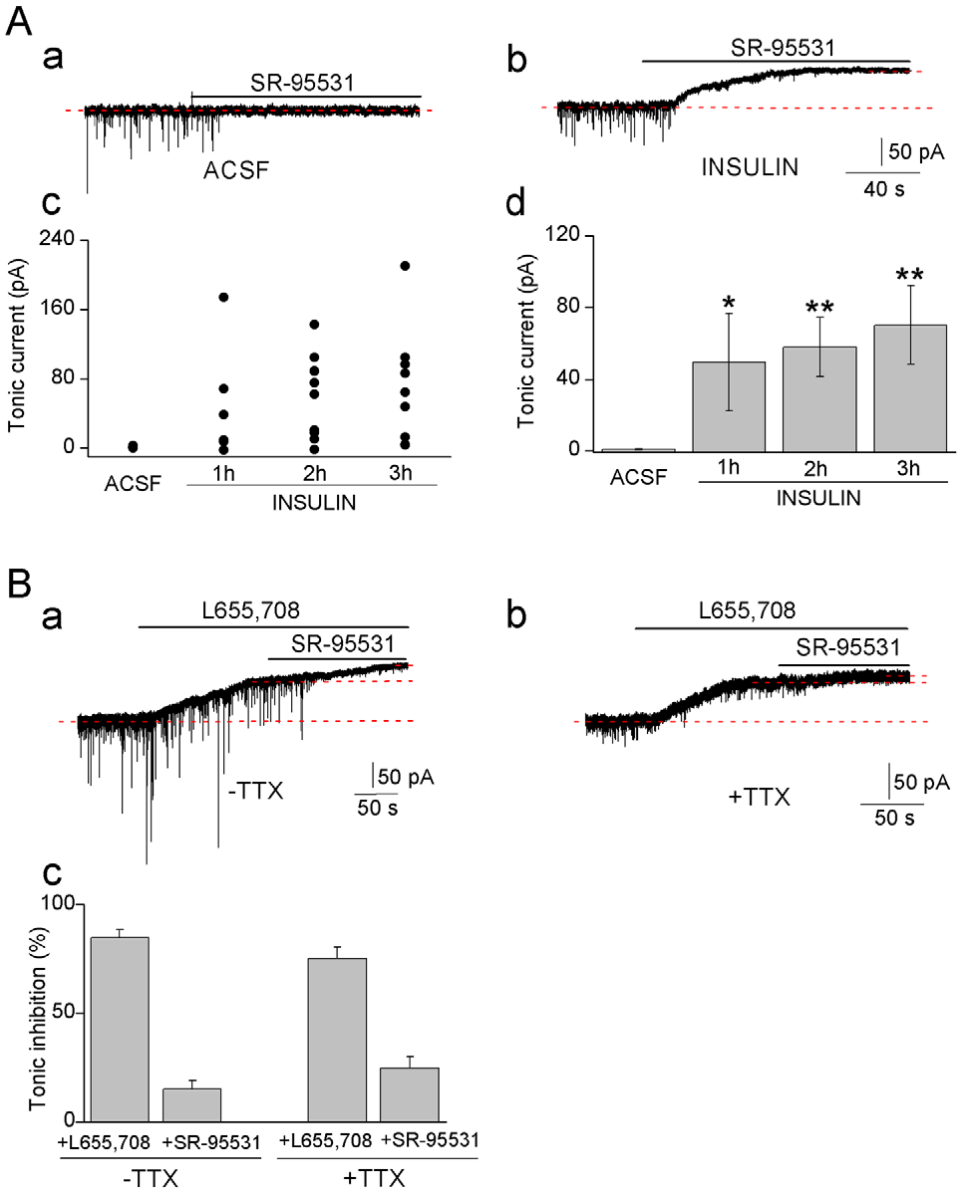
Insulin (1 nM) induces tonic GABA_A_ currents in CA1 pyramidal neurons. **A**. An ACSF (**a**) and an insulin-treated neuron (**b**, 2 h), SR-95531 (20 µM). **c**, Amplitudes of tonic currents recorded in individual neurons; ACSF (n = 11), incubated with insulin 1 h (n = 6), 2 h (n = 9), 3 h (n = 9). **d**, The averaged tonic current amplitudes, mean ± SEM. * *P*<0.05, **P<0.01. **B** Inhibition of insulin induced tonic current by L655, 708 (5 µM) in the absence (**a**) and presence (**b**) of TTX (1 µM) and a GABA_B_ antagonist CGP 52432, SR-95531 (20 µM). **c**, % of total tonic current inhibited. ns, no significance.

The whole-cell currents were recorded from rat hippocampal CA1 pyramidal neurons. After applying SR-95531 (20–200 µM), a GABA_A_ channel antagonist, to control slices bathed in artificial cerebrospinal fluid (ACSF slices), the spontaneous inhibitory postsynaptic currents (sIPSCs) were blocked but the holding current did not or only shifted marginally indicating no or minor tonic currents activated in the neurons (Fig. 1Aa) and is in accordance with previous reports [8], [9], [18], [19]. In contrast, in slices incubated with insulin, a clear shift of the holding current was observed (Fig. 1Ab) revealing a tonic current induced by insulin. It has been shown that 500 nM insulin increases miniature IPSCs (mIPSCs) amplitudes in pyramidal neurons [17] but effects on tonic currents have not been reported. We examined a range of insulin concentrations (0.5–100 nM) for their ability to induce tonic currents in the CA1 pyramidal neurons. Only 0.5 nM insulin failed to consistently induce tonic currents in neurons (50% of trials, n = 8). In slices incubated with 1nM insulin in the presence of wortmannin (100 nM), an inhibitor of a key enzyme phosphoinositide 3-kinases (PI3Ks) in the insulin receptors intracellular cascade, no induced tonic current was detected (n = 8, data not shown). As insulin at concentrations greater than 1 nM can stimulate both the insulin receptors and insulin-like growth factor-I receptors (IGF-IR) [20], we incubated the hippocampal slices with 1 nM insulin in this study. One nM insulin is also within the physiological range [4] and crosses the blood-brain barrier by a saturable transport mechanism [21]. Tonic current levels recorded in the ACSF control and slices treated with insulin for 1–3 h are shown in Fig. 1 Ac and d. In 22 of a total of 24 neurons tested did insulin induce tonic currents and the averaged currents were similar at 1, 2 and 3 hrs (49.5±27.1, 58.0±16.4, 70.3±21.9 pA) and significantly larger than in the control slices (0.7±0.3 pA).

### Insulin induced tonic current is carried by α5, γ2 containing GABA_A_ channels

In hippocampal CA1 pyramidal neurons either extrasynaptic α5 or δ containing GABA_A_ channels have been shown to carry the small tonic current that may be present in the neurons at basal ambient GABA concentrations [8], [9], [18], [19], [22] and increases when the extracellular GABA concentration is elevated by external applications of GABA [9], [14], [18]. We examined whether the insulin induced tonic current was inhibited by the GABA_A_ inverse agonist L655, 708 (5 µM) that is selective for channels containing the α5 and γ2 subunits in the channel complex (Fig. 1B). In the insulin treated slices, L655, 708 inhibited the holding current (85±4%) causing an upward shift of the current level and co-application of SR-95531 further inhibited the current (15±4%) (Fig. 1Ba, b, c). This demonstrates that the insulin induced current is at least partially carried by α5βγ2 GABA_A_ channels (Fig. 1Bc). We repeated the experiments in the presence of the sodium channel blocker TTX (tetrodotoxin, 1 µM) to inhibit action potential-dependent synaptic activity and examined whether the large tonic current observed after insulin incubation of the slices was related to increased synaptic activity, resulting in higher extracellular GABA concentrations from synaptic spillover of GABA and thereby activating a larger proportion of the relatively low-affinity (EC_50_>1 µM) GABA_A_ channels that are present in the neurons [23]. Interestingly, the average amplitude of the sIPSCs was decreased from 37.8±1.4 pA to 21.4±0.4 pA (n = 5) but the induced tonic current remained (Fig. 1Bb, c) further suggesting that the newly turned-on GABA_A_ channels are more sensitive to the ambient GABA, determined by e.g. GABA transporters activity or synaptic spillover, than the ones normally expressed.

### Insulin-induced GABA_A_ tonic currents are inhibited by flumazenil and zolpidem

**Figure 2:**
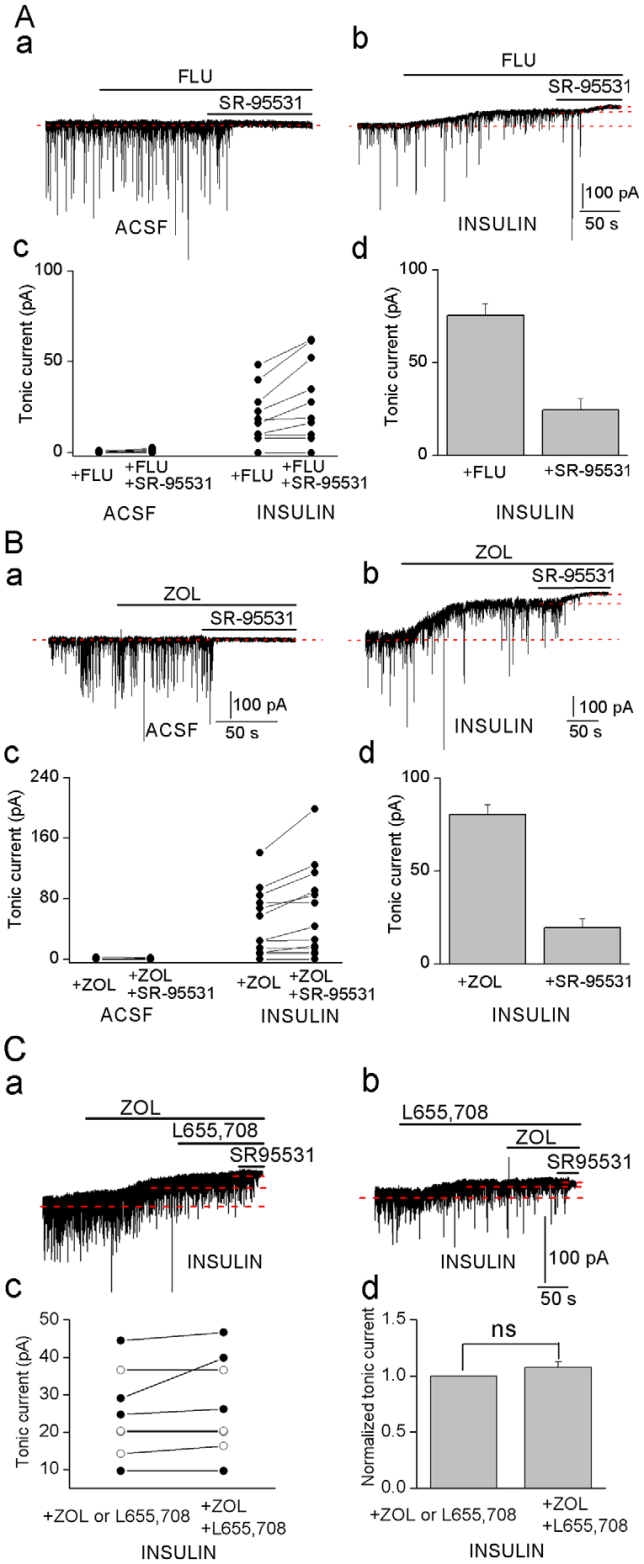
Inhibition of insulin (1 nM) induced tonic current by flumazenil and zolpidem. Effects of flumazenil (**A**, 1 µM), zolpidem (**B**, 100 nM) and SR-95531 (20 µM) on the currents in an ACSF (**a**) and an insulin treated neuron (**b**). **c**, Amplitudes of tonic currents in individual neurons; **A**: ACSF (n = 8), insulin incubated (n = 10); **B**: ACSF (n = 6), insulin-incubated neurons (n = 13). **d**, A & B, % of total tonic current inhibited by antagonists. **C**. Tonic currents (**a**, **b**) in the presence of L655, 708 (5 µM) or zolpidem (100 nM) or the drugs together (L655, 708,5 µM; zolpidem, 100 nM) in insulin treated neurons. **c**. Amplitudes of the tonic currents in individual neurons (L655, 708 first applied alone: open circle, n = 4; zolpidem first applied alone: filled circle, n = 4). **d**. Tonic current inhibited by L655, 708 plus zolpidem (n = 8) normalized to the tonic current inhibited by either L655, 708 or zolpidem in the same cell. ns, no significance.

**Figure 3:**
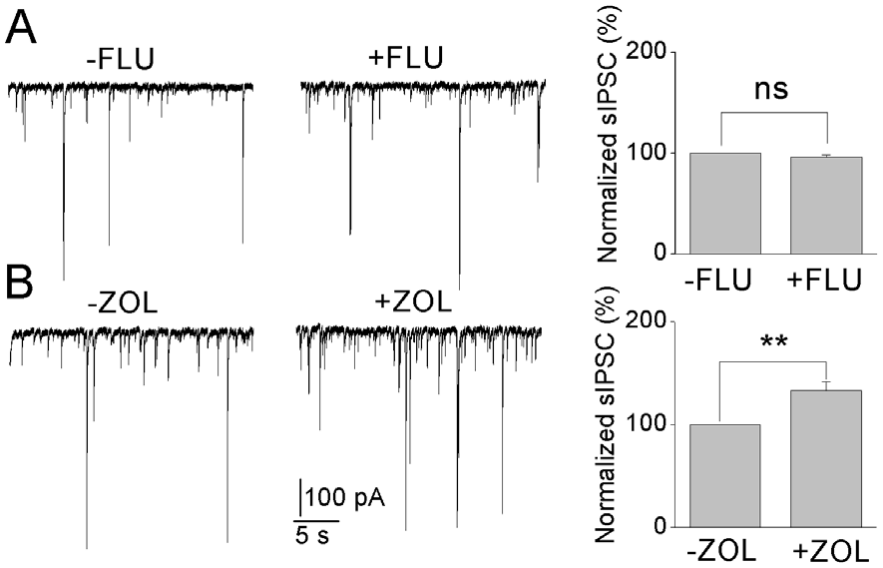
sIPSCs recordings in insulin-incubated neurons recorded before (−) and after (+) application of (A) flumazenil (1µM), (B) zolpidem (200 nM) and total sIPSCs normalized to before flumazenil or zolpidem application, mean ± SEM, n = 6. ** *P*<0.01.

GABA_A_ channels are pentamers formed by subunits from 8 different families (α1–6, β1–4, γ1–3, δ, ε, θ, π, ρ1–3). The channel is thought to be composed of two αs, two βs and a third subunit type that is often the δ or the γ2 subunit [24]. Whether the native channels are homo or heteromeric in terms of the αs and βs subunits is not clear [25]. We examined two compounds that are normally inert at α5βγ2 channels: the benzodiazepine flumazenil (1 µM) and zolpidem (100 and 200 nM), a positive modulator at α1, α2 and α3 in αβγ2 channels. Surprisingly, in slices incubated in insulin both flumazenil (Fig. 2A) and zolpidem (Fig. 2B) shifted the holding current revealing the large insulin-induced tonic current. Similar to L655, 708, flumazenil and zolpidem inhibited the tonic current by 76±6% and 81±5%, respectively (Fig. 2). Flumazenil and zolpidem had minimal effect on the holding current level in the control slices (Flumaznil, n = 8 and zolpidem, n = 6, Fig. 2A & B) consistent with other studies [14], [26]. We examined if L655, 708 and zolpidem affected the same population of channels by first applying one and then the two drugs together and examined the shift in the holding current (Fig. 2C). The results show that it does not matter in which order the drugs are applied, the effects are not additive. Rather, it appears that the first drug applied occludes the effect of the other consistent with that the drugs are acting on the same population of channels. At synapses, flumazenil is normally inert whereas zolpidem is known to potentiate the currents [14], characteristics that are maintained in the insulin incubated slices (Fig. 3A & B).

### Insulin-induced tonic conductance decreases the excitability of CA1 pyramidal neurons

**Figure 4:**
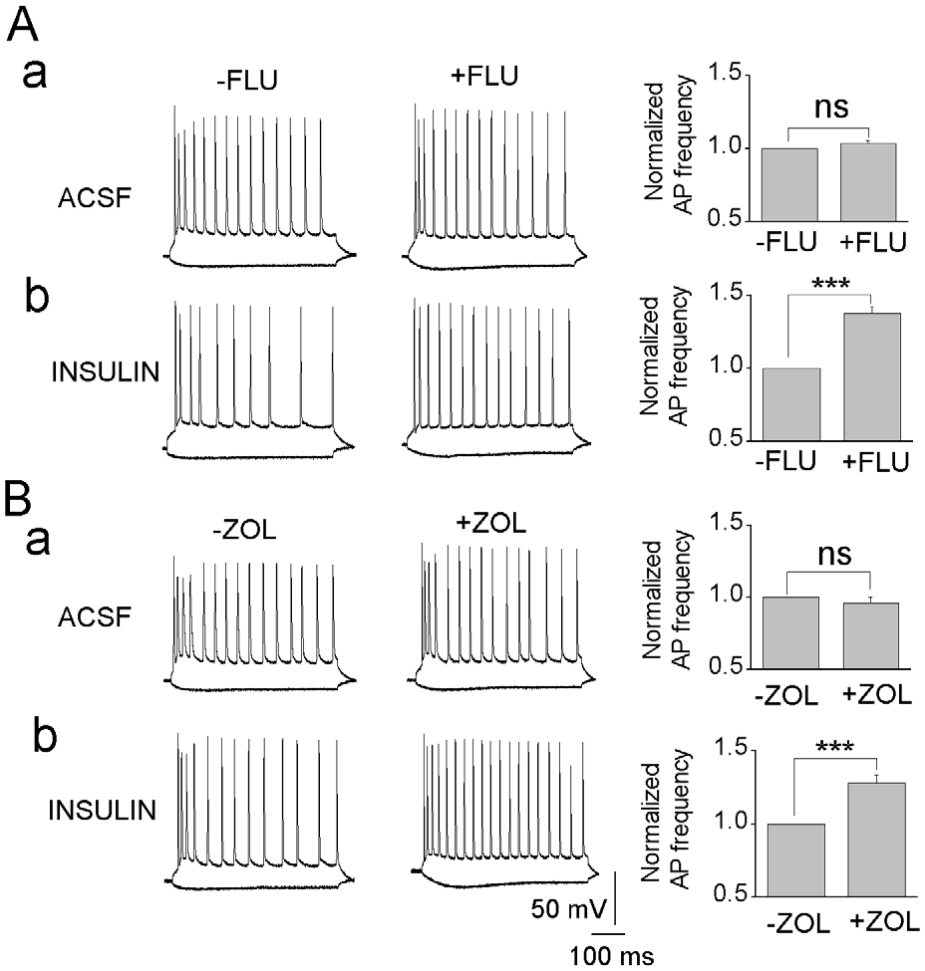
Flumazenil and zolpidem increase the excitability of insulin-treated CA1 pyramidal neurons by inhibiting tonic GABA_A_ channels. Current-clamp traces illustrate action potential (AP) firing evoked by a 100 pA current step (500 ms) in ACSF (**a**) and insulin-incubated neuron (**b**) before and after application of flumazenil (**A**, 1 µM) or zolpidem (**B**, 100 nM). Bar graphs; ACSF (FLU: n = 4; ZOL: n = 4), insulin incubated neurons (FLU: n = 6; ZOL: n = 7). **, P<0.001.

GABA-mediated tonic inhibitory conductances have been shown to decrease excitability of neurons by modulating the firing frequency of action potentials [15]. The CA1 pyramidal neurons in slices incubated with insulin had on the average resting membrane potential of −59.2±0.4 mV (n = 10) whereas in control slices it was −57.6±0.4 mV (n = 6) and accordingly, we found that insulin incubation of the slices significantly decreased the action potential firing rate (23.2±1.2 Hz, n = 10) as compared with ACSF control (27.7±1.6 Hz, n = 6). Since flumazenil and zolpidem inhibit the tonic current induced by insulin these drugs can be expected to modulate the firing frequency of the CA1 neurons. Fig. 4A and B show that both 1 µM flumazenil and 100 or 200 nM zolpidem, respectively, significantly increase the action potential firing rate in insulin-treated but not in ACSF control neurons.

### New, high-affinity GABA_A_ channels are turned-on by insulin

**Figure 5:**
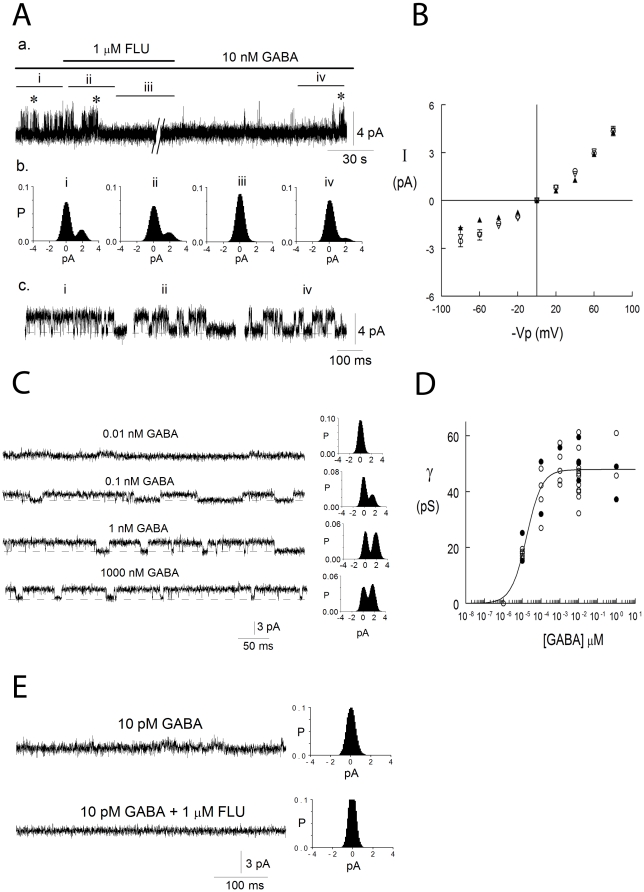
GABA activates single-channel currents in a graded manner and the currents are inhibited by flumazenil. **A**. 10 nM GABA-activated channels in a cell-attached (ca) patch that was then ripped-off the cell and an inside-out (io) patch formed (−Vp = 40 mV). **a**, In a reversible manner 1 µM flumazenil inhibits the GABA-activated current. **b**, 30 s (i, ii, iv) and 3.5 min (iii) all-points open probability histograms **c**, Currents from the trace shown in **a** (*) **B**. Average IV relation, currents activated by 10 nM GABA in ca (○, open circle, n = 5–18), io (▽, open triangle, n = 3–6) patches. ▴, filled triangle, an io patch were currents were later inhibited by 1 µM flumazenil. **C**. GABA-activated currents in ca patches (−Vp = 40 mV) and 10 s all-points open probability histograms. 0.01, 0.1, 1 and 1000 nM GABA activated 0.6, 1.3, 2.2 and 2.0 pA channels, respectively. All patches were later made io and currents inhibited by 1 µM flumazenil. **D**. Conductance-GABA concentration relation, 52 ca patches, −Vp = 40 mV. •, patches later made io and currents blocked with 1 µM flumazenil. E. 10 pM GABA-activated channels in a ca patch that was then ripped-off the cell and an io patch formed (−Vp = 40 mV). The recordings are from the io patch in 10 pM GABA in the absence and presence of 1 µM flumazenil and the all-points histograms are each from a 10 s current trace that includes the sample trace shown.

The average extracellular concentration of GABA has been recorded to be about 25 nM in the dentate gyrus of the hippocampus [10] where it activates high-affinity extrasynaptic GABA_A_ channels [27]. Under our experimental conditions, no extracellular GABA was added and thus the tonic current must have been activated by GABA concentrations existing within the slice. We therefore examined in insulin-incubated slices if we could activate GABA_A_ channels using similar GABA concentrations (10 nM) and then block these channels with flumazenil. The results in Fig. 5 show channels that were first activated in intact neurons in the cell-attached (ca) configuration. The patch was then ripped off the cell and flumazenil was applied to the bath. We have previously shown that benzodiazepines applied to the intracellular phase of the cell membrane can cross the bilayer and modulate channels from the extracellular side [26]. Fig. 5A shows 10 nM GABA-activated 45 pS channel (−Vp = 40 mV, Fig. 5A ai, bi, ci) that was then inhibited by 1 µM flumazenil (Fig. 5A aiii, biii) and remained closed until flumazenil was washed off (Fig. 5A aiv, biv, civ). The channels showed outward rectification as shown by the current-voltage relationship of a channel (▴, filled triangles, Fig. 5B) that was later blocked by 1 µM flumazenil. Similar IV curves were recorded both in intact cells (○, open circles, n = 18) and ripped-off patches (▽, open triangles, n = 6). We then examined what GABA concentrations could activate channels in the intact CA1 pyramidal neurons and the results are shown in Fig. 5C and D (−Vp = 40 mV). Concentrations as low as 10 pM could activate the channels and the average maximal-current amplitude was reached with 1 nM GABA. The all-points histograms in Fig. 5C are from 10 s current recordings and show the gradual increase in single-channel current amplitude with increased GABA concentration. All patches were later made inside-out and the currents blocked with 1 µM flumazenil. In Fig. 5D single-channel conductances from 52 cell-attached patches at depolarized 40 mV (−Vp = 40 mV) are plotted as open circles and then those that were later blocked by 1 µM flumazenil as filled circles. The data was fitted by a Hill-type equation (see methods) and gave the half-maximal GABA concentration for activation (EC_50_) of 17±4 pM, I_max_ of 48±1 pS and a Hill coefficient of 1.0±0.2. The ten pM GABA concentration activated channels with the average maximum-conductance of 18±2 pS (n = 8) and a noise baseline holding current (Fig. 5E). In the presence of 1 µM flumazenil the channels were inhibited and the holding current baseline noise decreased as can be seen by comparing the current traces before and after the addition of flumazenil and evidenced by the narrowing of the all-points histogram in the presence of the flumazenil. The results are consistent with GABA activation of channel subconductance states at even the low 10 pM GABA concentration. These channels have by far the highest affinity for GABA of any described GABA_A_ channels and are activated by about 10∧7 lower GABA concentrations than synaptic channels.

## Discussion

Our results demonstrate that in hippocampal CA1 neurons, physiological concentrations of insulin induce tonic conductance that is generated by novel, high-affinity GABA_A_ channels and when in place, regulates the CA1 neurons excitability.

There appears to be numerous ways in which GABA mediated tonic inhibition may arise; it can be activated by (1) the ambient level of GABA around the neurons [9], [10], [23], [27], [28], [29], (2) by increased extracellular GABA concentrations by mechanisms such as spillover of GABA from synapses or nonvesicular release of GABA [18], [23], [28] or (3) as we have shown in this report, by insulin which induces new high-affinity extrasynaptic receptors that can sense the ambient level of GABA. If the ambient level of GABA in the CA1 hippocampal region is similar to what it is in the dentate gyrus (25 nM) then the new channels with an EC_50_ of 17 pM will be saturated with GABA. In effect, insulin then acts as a switch to turn-on tonic inhibition in the CA1 pyramidal neurons. As the channels show outward rectification they are expected to predominantly affect neurons at the spiking threshold [15] and accordingly in our study the insulin-induced tonic conductance decreased frequency of action potential firing in the CA1 pyramidal neurons.

Different GABA_A_ channel assembles containing α1, α5, α4, α6, γ2, δ or ε subunits have been shown to mediate the tonic conductance in CNS neurons [23], [27], [28], [29], [30]. In our study, the insulin-induced tonic current is mainly carried by α5, γ2 containing GABA_A_ channels. GABA_A_ channels having the α5 subunit in their channel complex are known to be mostly located extrasynaptically in CA1 neurons but are not or minimally activated by the ambient GABA concentration. How the new channels differ from the α5-channels normally in the membrane is not clear but heteromeric α subunits in the channel complex, different intracellular modification or associations with intracellular proteins can all give rise to the differences observed [24], [25]. Interestingly, the induced tonic current is inhibited by flumazenil and zolpidem, indicating a distinct pharmacology of these novel GABA_A_ channels. In the presence of zolpidem there was a significant increase in the action potential firing rate in insulin-treated but not in ACSF control neurons. These results are somewhat surprising as zolpidem potentiates the synaptic currents and its effects on the tonic current would at least partially be cancelled by the increased sIPSCs. Since the overall effect of zolpidem in the insulin treated slices was increased excitability of the neurons, it supports the notion [15], [28], [31] that tonic rather than synaptic conductances regulate basal neuronal excitability when significant tonic conductance is expressed.

Decline in cognitive abilities is associated with a number of diseases including Alzheimer disease, dementia and diabetes mellitus. These diseases already affect a large proportion of populations worldwide and are increasing in prevalence. We have identified a specific target in the hippocampus, a new subtype of GABA_A_ channels turned-on by insulin that may potentially prove useful when rescuing cognition in these folk diseases.

## Materials and Methods

### Hippocampal slice preparation

Experiments were performed on hippocampal slices from postnatal 16–22 days old Wistar rats. The animals were sacrificed in accordance with local ethical guidelines and approved animal care protocols by the Uppsala Djurförsöksetiska Nämnd, Sweden (Uppsalas' animal ethics committee) and the study given the approval ID C258/8. Hippocampal slices were prepared as previously described [6]. In brief, the rat brain was quickly removed and sagittal hippocampal slices (400 µm thick) were cut with a vibratome (Leica VT1200) in an ice-cold artificial cerebrospinal fluid (ACSF) containing (mM): 124 NaCl, 26 NaHCO_3_, 3 KCl, 1.3 MgSO_4_, 2.5 Na_2_HPO4, 2.5 CaCl_2_ and 10 glucose with pH 7.2–7.4 when bubbled with 95% O_2_ and 5% CO_2_. Slices were incubated in the same ACSF solution at 37°C for 1h and then stored at room temperature (20–22°C). Insulin (0.5–100 nM) was dissolved in ACSF and applied to the slices for 1–3 hours.

### Electrophysiological recording and analysis

All patch-clamp recordings were performed at room temperature (20–22°C). All drugs used were purchased from Sigma-Aldrich or Ascent Scientific. SR-95531 was dissolved in the bath solution. L655, 708, flumazenil and zolpidem were dissolved in DMSO [26] to generate a stock solution that was later diluted in the bath solutions to give the final concentration.

For the voltage-clamp recordings slices were perfused with ACSF containing kynurenic acid (3 mM) and other drugs and equilibrated with 95% O_2_ and 5% CO_2_. The pipette solution containing (mM): 140 CsCl, 1CaCl_2_, 3 EGTA, 0.5 KCl, 1 MgCl_2_, 2 ATP-Mg, 0.3 GTP-Na, 5 QX-314 bromide, 10 TES, pH of 7.25 with CsOH. Recordings were made at −60 mV and rejected for analysis if the access resistance changed more than 25%. For the current-clamp recordings the slices were perfused with ACSF and the pipette solution contained (in mM): 130 K-gluconate, 5 NaCl, 2 MgCl_2_, 0.5 EGTA, 2 ATP-Mg, 0.3 GTP-Na, 10 HEPES, pH 7.25 with NaOH. Single-channel recording solutions were: chamber, mM: 140 NaCl, 5 KCl, 1 MgCl_2_, 1.8 CaCl_2_ and 10 TES, pH 7.25; pipette, mM: 140 Choline Cl, 5 NaCl, 1 KCl, 1 MgCl_2_, 1.8 CaCl_2_, 10 TES, pH 7.25, 200 µM saclofen and 0–1 µM GABA. Electrodes were made from borosilicate glass and the resistance was 2–4 MΩ (whole-cell recording) or 10–20 MΩ (single-channel recording) when filled with the respective pipette solution.

Patch-clamp recordings were done using an Axopatch 200B amplifier, filtered at 2 kHz, digitized on-line at 10 kHz using an analogue-to-digital converter and analyzed with pClamp (Molecular Devices) and MiniAnalysis (Synaptosoft) programs. The amplitude of the tonic current was measured as the difference in the holding current level before and after drug application [8]. 50–150 spontaneous inhibitory postsynaptic currents (sIPSCs) events were randomly selected from each recording and total sIPSCs calculated according to f×Q, f is the frequency of sIPSCs and Q is the average value for charge transfer per sIPSC [8]. The amplitude of single-channel currents was measured from peaks in all-points histograms or by direct measurements of the single-channel current amplitude. A Hill-type equation was fitted to the single-channel data: γ = γ_max_ * [GABA]^n^/[EC_50_]^n^+[GABA]^n^. Statistical comparisons used unpaired student t-test or one-way ANOVA.
